# Projected impacts of climate change on regional capacities for global plant species richness

**DOI:** 10.1098/rspb.2010.0120

**Published:** 2010-03-24

**Authors:** Jan Henning Sommer, Holger Kreft, Gerold Kier, Walter Jetz, Jens Mutke, Wilhelm Barthlott

**Affiliations:** 1 Nees Institute for Biodiversity of Plants, University of Bonn, Meckenheimer Allee 170, 53115 Bonn, Germany; 2 Biodiversity, Macroecology and Conservation Biogeography Group, University of Göttingen, Büsgenweg 2, 37077 Göttingen, Germany; 3 Department of Ecology and Evolutionary Biology, Yale University, 165 Prospect Street, New Haven, CT 06520, USA

**Keywords:** biodiversity patterns, global warming, water-energy dynamics, water-energy-richness hypothesis

## Abstract

Climate change represents a major challenge to the maintenance of global biodiversity. To date, the direction and magnitude of net changes in the global distribution of plant diversity remain elusive. We use the empirical multi-variate relationships between contemporary water-energy dynamics and other non-climatic predictor variables to model the regional capacity for plant species richness (CSR) and its projected future changes. We find that across all analysed Intergovernmental Panel on Climate Change emission scenarios, relative changes in CSR increase with increased projected temperature rise. Between now and 2100, global average CSR is projected to remain similar to today (+0.3%) under the optimistic B1/+1.8°C scenario, but to decrease significantly (−9.4%) under the ‘business as usual’ A1FI/+4.0°C scenario. Across all modelled scenarios, the magnitude and direction of CSR change are geographically highly non-uniform. While in most temperate and arctic regions, a CSR increase is expected, the projections indicate a strong decline in most tropical and subtropical regions. Countries least responsible for past and present greenhouse gas emissions are likely to incur disproportionately large future losses in CSR, whereas industrialized countries have projected moderate increases. Independent of direction, we infer that all changes in regional CSR will probably induce on-site species turnover and thereby be a threat to native floras.

## 1. Introduction

Global climate has been warming by approximately 0.6°C during the last three decades (Hansen *et al*. 2006). The bulk of warming observed over the last 50 years can be with high certainty attributed to human-induced greenhouse gas emissions (Raupach *et al*. 2007). Global warming is expected to continue at ever growing rates in the current century, and some scenarios indicate a global temperature rise by up to 6°C by 2100 (IPCC 2007; Richardson *et al*. 2009).

There is compelling empirical evidence that climate change affects life on Earth in many ways. Prominent examples are phenological characteristics like flowering time of plants (Walther *et al*. 2002; Parmesan 2006; Høye *et al*. 2007), breeding and arrival of migratory species (Both & Visser 2001; Walther *et al*. 2002) and already, evolutionary adaptations to the changing conditions have been recorded (Bradshaw & Holzapfel 2006). Climate change also influences species distributions and richness as well as the composition of assemblages (Parmesan & Yohe 2003; Root *et al*. 2003; Thuiller *et al*. 2008). Species may either keep their current range or respond to changing environmental conditions with range expansions, contractions or shifts. Colonization of new suitable areas may result in poleward or upslope range expansions (Walther *et al*. 2002; Parmesan 2006). Retractions from unsuitable sites with harsher environmental conditions may lead to local and even global extinction events (Thomas *et al*. 2004; Thuiller *et al*. 2005). As a consequence, the warming can provoke a lowland biotic attrition in some diverse tropical lowland areas (Colwell *et al*. 2008). Altogether, these processes generate local and regional turnover of species and net changes in species richness (Peterson *et al*. 2002).

Consequences of changing climatic conditions on the size and location of species ranges can be calculated by applying niche modelling that takes into account information on habitat requirements derived from known occurrence sites (Guisan & Zimmermann 2000; Scott *et al*. 2002; Phillips *et al*. 2006). Such models provide descriptors of species' habitat and environment space that can then be applied to future climate scenarios (Sykes *et al*. 1996; Midgley *et al*. 2002; Pearson & Dawson 2003; Skov & Svenning 2004; McClean *et al*. 2005; Thuiller *et al*. 2005; Araújo & Rahbek 2006; McKenney *et al*. 2007). Despite the fact that these models indicate potential rather than realized distributions, the difference between recent and potential future distribution provides valuable information on possible range shifts (Guisan & Thuiller 2005), the risk status of current ranges and required dispersal rates to reach new suitable habitats (Jump *et al*. 2009). Plant distribution datasets have been assembled and analysed at regional to continental extents (e.g. Linder 2001; Crisp *et al*. 2001; Thuiller *et al*. 2005; Küper *et al*. 2006; Jiménez *et al*. 2009), but comparative analyses of these datasets at a global scale remain intractable owing to their uneven taxonomic and geographical representation (Yesson *et al*. 2007). As an alternative to species distribution data, information on species richness for operational geographical units can be used for mapping of geographical patterns of plant diversity (Barthlott *et al*. 2005; Mutke & Barthlott 2005), for establishing environment-richness relationships (Ricklefs *et al*. 2004; Kreft & Jetz 2007) or for modelling future changes (Algar *et al*. 2009).

Species richness, the number of species per area, is strongly affected by climatic constraints, and water-energy relations are the strongest and most pervasive predictors of broad-scale gradients of plant species richness among different environmental variables (O'Brien 1998; Hawkins *et al*. 2003; Currie *et al*. 2004; Field *et al*. 2005; Kreft & Jetz 2007). The water-energy-richness hypothesis has received ample empirical support (Field *et al*. 2009). It states that at high latitudes, plant species richness is more strongly controlled by ambient energy, whereas at low latitudes, the availability of liquid water becomes more important (Hawkins *et al*. 2003). Vascular plants most probably evolved under wet tropical conditions (Crane & Lidgard 1989) and needed to expand their niche breadth by developing additional adaptations of survival under less-favourable climate conditions (Wiens & Donoghue 2004). Hence, the potential distribution of species is mostly constrained by their physiological level of tolerance, for example, their ability to deal with frost and drought (O'Brien 1998; Hawkins *et al*. 2003; Currie *et al*. 2004). Moreover, biotic interactions influence the realized ranges of species (Araújo & Pearson 2005; Soberón 2007). In addition to climatic controls, topography and habitat heterogeneity also affect the species richness of an area. Especially in warmer climates, topographically diverse regions have a generally higher potential to maintain high species numbers (e.g. Kerr & Packer 1997; Kreft & Jetz 2007; Jiménez *et al*. 2009).

While climate and other environmental variables are strong predictors of species richness, recent studies have shown significant differences in the species richness of different biogeographical regions after controlling for these effects (Kreft & Jetz 2007; Qian 2009). For plants, prime examples are winter rainfall regions that have higher richness than expected from their current climate (Cowling *et al*. 1996; Linder 2001). This suggests that idiosyncratic regional events as well as long-term climate fluctuations play an additional role in shaping species-richness patterns (Dynesius & Jansson 2000).

Different approaches describing water-energy dynamics have been successfully used in mechanistic or correlative global models of contemporary plant diversity (Kleidon & Mooney 2000; Francis & Currie 2003; Sitch *et al*. 2003; Venevsky & Venevskaia 2003; Kreft & Jetz 2007). It has been demonstrated that these associations may be used to estimate potential effects of climate change and atmospheric carbon dioxide concentrations on broad-scale species-richness patterns (Currie 2001; Woodward & Kelly 2008; Algar *et al*. 2009). Such correlative approaches ignore obvious long-term evolutionary processes (Fine & Ree 2006; Donoghue 2008), but facilitate basic estimates that would otherwise not be feasible at a broad scale (within relevant time frames) in such mega-diverse groups as plants. Here, we seize on the potential of this methodology and model for present and future, at a global scale, the number of species for which a region can potentially provide habitat space. As this potential species number is based purely on the environmental capacity per area and may therefore differ from the actual species number, it is denominated ‘regional capacity for species richness’ (CSR).

## 2. Material and methods

We apply an empirical, correlative approach to relate the observed global variation in plant species richness to a set of ecologically meaningful environmental predictors (see details below) in a generalized linear modelling (GLM) framework using the software package R (R Development Core Team 2005). Hawkins *et al*. (2003) and Kreft & Jetz (2007) showed that variables related to ambient energy are stronger determinants of species richness in high-latitude regions with low temperatures, whereas in areas with warmer temperatures (at lower latitudes), availability of water is more important. Accordingly, our study follows this ‘water-energy-richness hypothesis’. The different climate change simulations imply shifts in the water-energy budget of an area, and the shifts are likely to affect CSR. In all cases, a consistent relationship is assumed between future climate predictors and CSR, and the model was likewise applied for regions holding combinations of future climate parameters that have no recent equivalent.

### (a) Species-richness data

We selected 1032 globally representative, non-overlapping natural and political operational geographical units with defined locality and with known or estimated plant species numbers from an exhaustive and geographically representative literature database (for more details and bibliographic information, compare Kier *et al*. 2005). These had been earlier compiled to map and analyse the contemporary distribution of global plant diversity (Barthlott *et al*. 2005; Kreft & Jetz 2007). Median area size was 22 910 km^2^, ranging from 13.49 to 575 400 km^2^, and we excluded small oceanic islands because environment-richness relationships differ between islands and mainlands (Kreft *et al*. 2008).

### (b) Climate datasets

As reference for possible future climate change, we used the different families of twenty-first century greenhouse gas emission scenarios selected by the Intergovernmental Panel on Climate Change (IPCC 2000), which are based on certain assumptions on technological and socio-economic development pathways and policy options (A1FI, A2, B1, B2). For the main comparative analyses, we referred to the two extreme cases A1FI and B1. The climate dataset comprises one contemporary (mean values for reference period 1960–1990, here referred to as ‘today’) and 18 future climate datasets for 2100 referring to five general circulation models (GCMs), i.e. CGCM2, CSIRO2, ECHAM4 (only A2/B2), HadCM3 and PCM in all combinations of the four major IPCC scenarios, distributed by the Tyndall Center for Climate Change Research (TYN SC 2.03 dataset; see Mitchell *et al*. 2004). The fossil-intensive A1FI scenario (IPCC 2000) results in a best estimate of an average global surface temperature rise of +4.0°C by 2100. By contrast, the technology-oriented B1 scenario results in a respective temperature rise of +1.8°C. However, there is growing evidence that the continuation of the current development of anthropogenic CO_2_ emissions would even result in a possible temperature rise above the A1FI scenario (Richardson *et al*. 2009).

### (c) Predictor variables and modelling

As environmental predictors in the GLM framework, we derived six variables. The four non-climatic variables were identical to those used in a similar model of global plant species diversity (Kreft & Jetz 2007), but two climate-related variables were slightly different. Potential evapotranspiration was replaced by mean annual temperature (log_10_ transformed; K) as an ambient energy-related predictor, because it is the major and most robust variable derived from future GCMs. Further, wet-day frequency was replaced by water balance, an alternative proxy for the water availability, because there were no data on wet-day frequency available from future climate surfaces. Water balance was calculated as the amount of precipitation minus the potential evapotranspiration per area (log_10_ transformed; mm yr^−1^), following the Thornthwaite equation (an approximation incorporating temperature and day length; see Thornthwaite 1948). We did not fit the complex inter-annual variation of these parameters. All other variables were identified as best predictors from a set of 40 analysed variables and were described in detail in Kreft & Jetz (2007). They are: area size (to control for the variation in the size of the operational geographical units; km^2^), habitat heterogeneity (measured as an index combining the number of elevational belts and vegetation types; *n*) and structural vegetation complexity (rank of three-dimensional complexity per biome ranging from one (desert, tundra) to six (tropical broadleaf forest); *n*). Additionally, to allow for differences across the superior biogeographical regions, floristic kingdom membership (*sensu* Olson *et al*. (2001) supplemented by the Cape Floristic Region (e.g. Takhtajan 1986); *n*) was included to account for regional effects on species richness above and beyond the environment (Kreft & Jetz 2007; Qian 2009). Acknowledging that climate-driven changes in land cover and vegetation structure might additionally affect future changes in CSR, the non-climatic variables were considered to remain constant, because reliable future projections of these parameters are not available and in order to analyse the individual contribution of water-energy dynamics to CSR changes. For the same reason, we did not consider land use changes or habitat integrity, even though these factors are known to heavily impact the distribution of species and may in many cases be the most relevant short- and medium-term threat to biodiversity (Sala *et al*. 2000; Jetz *et al*. 2007).

A six-predictor GLM was performed for global plant species richness and the combination of all abovementioned predictor variables. Additionally, the interaction between mean annual temperature and water balance was considered, following the hypothesis that the role of temperature to explain CSR may be different in areas with positive and negative water balance. The model parametrization was then used to predict CSR per standard area across a global equal area grid of *ca* 110 × 110 km^2^ (12 100 km^2^) for the current datasets. Assuming a consistent relationship between species richness and environment until 2100, the model parametrization derived from contemporary richness-environment relationships was then used to model future changes in CSR for 18 available combinations of the five GCMs and the four major IPCC scenarios. Then, the average values for each IPCC emission scenario were calculated as the mean of the respective GCMs. The main results presented here refer to these mean values of either the ‘optimistic’ B1 scenario or the ‘business as usual’ A1FI scenario.

## 3. Results and discussion

The six-predictor GLM explained 63.4 per cent of the deviance in current patterns of species richness based on two climatic and four non-climatic parameters (table 1). Compared with the model proposed by Kreft & Jetz (2007), this explained about 2 per cent less of the deviance, but yielded very similar estimates of species richness (*r*_s_ = 0.92).

**Table 1.** Generalized linear model (GLM) results of a model combining six predictor variables. (Since spatial autocorrelation might affect traditional statistical tests, we additionally performed spatial linear models to scrutinize *p*-values obtained from the GLM approach (spatial simultaneous autoregressive error model estimation, compare Kreft & Jetz (2007)). AREA, area size of operational geographical unit (km^2^); TMP, mean annual temperature (K); WB, water balance (mm yr^−1^); TMP : WB, interaction between TMP and WB; TOPOVEG, variable combining topographical complexity and number of vegetation types (*n*); STRUCT, structural complexity of vegetation (*n*); KINGDOM: NEA, Nearctic; AUS, Australis; CAP, Capensis; PAT, Paleotropic; PAA, Palaearctic; AIC, Akaike information criteria. Estimates for KINGDOM refer to deviations from the Neotropics (NET).)

	coefficient	s.e.	*t*	*p*
AREA	0.056	0.01	5.261	1.74 × 10^−7^
TMP (log)	−2847	350	−8.141	1.14 × 10^−15^
WB (log)	−1541	190	−8.106	1.49 × 10^−15^
TMP (log) : WB (log)	628	77	8.166	9.33 × 10^−16^
TOPOVEG	0.016	0.0008	19.234	<2 × 10^−16^
STRUCT	0.035	0.004	7.758	2.09 × 10^−14^
KINGDOM				
NEA	−0.054	0.031	−1.766	0.0776
AUS	−0.033	0.041	−0.797	0.4254
CAP	0.24	0.048	4.896	1.14 × 10^−6^
PAT	0.002	0.023	0.081	0.9358
PAA	−0.007	0.028	−0.237	0.8128
deviance, %	63.4			
AIC	−288.09			

**Figure 1.**
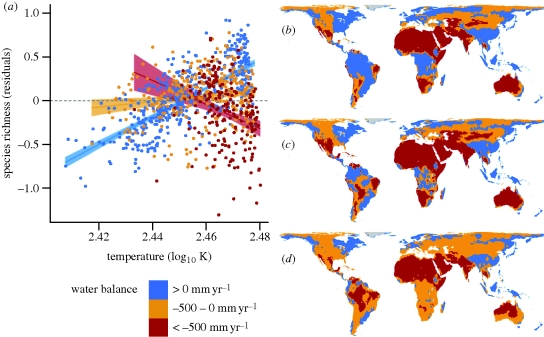
(*a*) Observed current effects of temperature on plant species richness in 1032 geographical units worldwide. Residuals from the species–area relationship (log–log) are plotted against log_10_ transformed mean annual temperature (in K) for three different classes of water balance (in mm yr^−1^) calculated as annual precipitation minus annual potential evapotranspiration per 110 × 110 km^2^ grid cell to illustrate the interaction effect between water balance and temperature. Regression lines with 95% confidence intervals are displayed for all three classes. (*b*–*d*) Global patterns of water balance. (*b*) Observed current patterns, (*c*) projected patterns under +1.8°C/B1 scenario for 2100, and (*d*) projected patterns under +4.0°C/A1FI scenario for 2100. Displayed are mean values for the CGCM2, CSIRO2, HadCM3 and PCM general circulation models (GCMs).

There was a strong interaction effect between temperature and CSR for different classes of water balance (figure 1*a* and table 1), and a model with an interaction between mean annual temperature and water balance provided stronger relative support than a model including only the main effects (*Δ*AIC = 106.5). In humid regions with positive water balance, there was a clear positive relationship between CSR and temperature (slope = 16.17 ± 0.65; standard error, *p* < 2 × 10^−16^). For regions with negative water balance up to −500 mm yr^−1^, this relationship was significantly shallower yet positive (slope = 3.16 ± 0.65, *p* = 0.006), and it was negative for the more arid areas with less than −500 mm yr^−1^ (slope = −13.82 ± 3.06, *p* = 1.05 × 10^−05^). As the water balance was predicted to get more negative in many regions according to the future climate scenarios (figure 1*b*–*d*), this leads to a predicted decrease in CSR in these regions.

**Figure 2.**
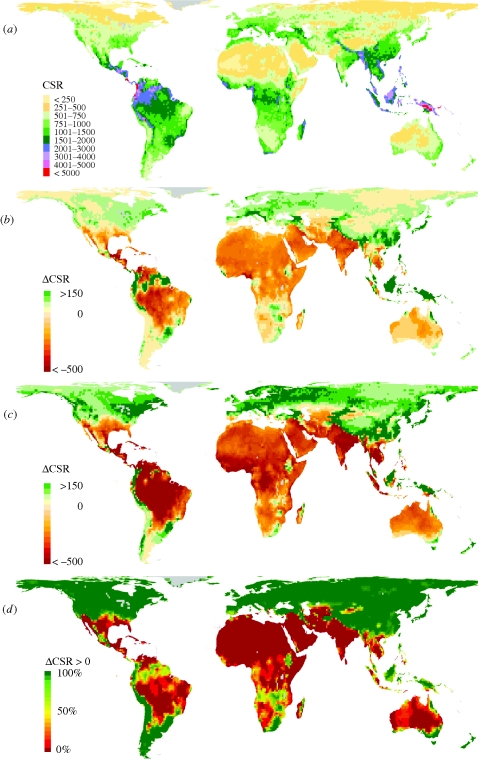
Modelled current global patterns of the capacity for species richness (CSR; species number per 110 × 110 km^2^) and future changes. (*a*) Modelled current patterns of CSR, (*b*) change in CSR under +1.8°C/B1 scenario for 2100, and (*c*) change in CSR under +4.0°C/A1FI scenario for 2100. CSR changes are counted in species numbers per 110 × 110 km^2^ grid cell and represent mean values for the CGCM2, CSIRO2, HadCM3 and PCM GCMs. Colour classes represent steps of 50 species. (*d*) Congruence in the direction of change (either increase in CSR or decrease in CSR, independent from the magnitude of change) between present and future CSR for all 18 available combinations of five GCMs (CGCM2, CSIRO2, ECHAM4 A2/B2, HadCM3 and PCM) and the four major IPCC scenarios (A1FI, A1, B1, B2). The dark green colour stands for 100% congruence across all 18 models that CSR is going to increase, whereas dark red indicates 100% congruence across models that CSR will decrease in the respective area. Yellow areas are subject to oppositional predictions of the direction of change across the models.

**Figure 3.**
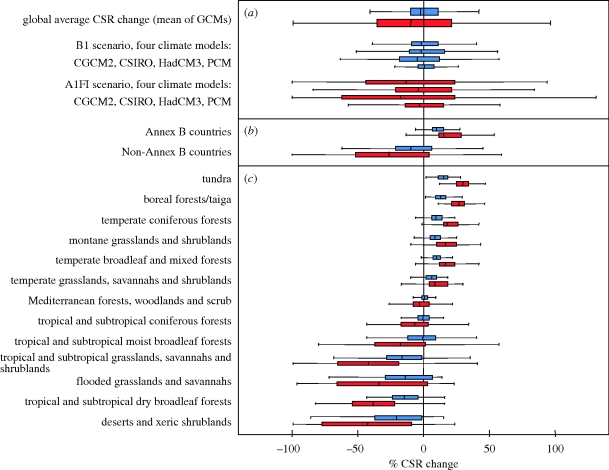
Modelled changes in the capacity for species richness (CSR; species number per 110 × 110 km^2^) between today and the year 2100 under the +1.8°C/B1 scenario (blue) and the +4.0°C/A1FI scenario (red). (*a*) Global average CSR change as mean values for the CGCM2, CSIRO2, HadCM3 and PCM GCMs, and for each GCM individually. (*b*) CSR change for the industrialized Kyoto protocol Annex B countries when compared with Non-Annex B countries. (*c*) CSR change across all 13 terrestrial biomes. Percentage values reflect the change in CSR for the respective subset of 110 × 110 km^2^ equal area grid cells. Bold lines indicate the mean value, boxes indicate second and third quartiles and whiskers indicate 10th and 90th percentiles.

The projected changes in future CSR relate to the magnitude of the projected temperature rise in a way that global average CSR declines stronger in scenarios with a higher expected temperature rise (figure 2 and table 2). The geographical distribution of future CSR per grid cell for the A1FI scenario differed significantly from the present (two-sample paired Wilcoxon signed-rank test, *p* < 2.2 × 10^−16^). This was not the case for the B1 scenario (*p* = 0.26, two-sample paired Wilcoxon signed-rank test, figure 2 and table 2). For the A1FI scenario, the CSR per grid cell was significantly lower than in the B1 scenario (figure 3*a*; two-sample paired Wilcoxon signed-rank test, *p* < 2.2 × 10^−16^), and individual CSR values per grid cell showed a higher variation (table 2). Global average CSR for the B1 scenario remained similar to the present when the mean of all GCMs was considered (+0.3%), but there were pronounced differences among them ranging from +3.0 per cent (PCM) to −2.9 per cent (HadCM3; table 2). For the A1FI scenario, there resulted a pronounced decrease in global average CSR with a mean decrease among all GCMs of −9.4 per cent, ranging from −0.7 (PCM) to −20.0 per cent (HadCM3; table 2).

**Table 2.** Summary results of future changes in the regional capacity for species richness (CSR; species number per 110 × 110 km^2^). (Presented are 18 combinations of four major IPCC emission scenarios (A1FI, A2, B1, B2) and five general circulation models (GCMs) (CGCM2, CSIRO2, ECHAM4 A2/B2, HadCM3, PCM) providing climate projections for the year 2100. *Global mean CSR change* (%) indicates the global average percentage change between current and future CSR across all grid cells. *Regional mean CSR change* (%) indicates the average absolute percentage change between current and future CSR as compared on an individual grid cell basis. *Global area with CSR loss* (%) gives the proportion of all grid cells that have lower values in future CSR than today. *Coeff. of variation in global CSR* displays the coefficient of variation as a normalized measure of dispersion of CSR, calculated as the ratio of the standard deviation of all regional CSR values to the global mean CSR. The higher the coefficient of variation, the more uneven is the distribution of regional CSR values.)

global mean CSR (today) = 887						
coeff. of variation in CSR (today) = 0.79		A1FI	A2	B1	B2	mean
CGCM2	global mean CSR change (%)	−15.6	−10.9	−0.3	−2.3	−7.2
	regional mean CSR change (%)	36.3	30.5	14.2	18.4	24.5
	global area with CSR loss (%)	53	51	40	44	50
	coeff. of variation in global CSR	0.95	0.94	0.90	0.91	0.91
CSIRO2	global mean CSR change (%)	−1.2	−3.3	1.7	0.5	−0.6
	regional mean CSR change (%)	27.2	30.5	19.7	22.7	24.9
	global area with CSR loss (%)	44	46	40	42	43
	coeff. of variation in global CSR	1.04	1.06	0.99	1.01	1.02
ECHAM4	global mean CSR change (%)	—	−12.0	—	−4.0	−7.9
	regional mean CSR change (%)	—	36.3	—	24.9	30.4
	global area with CSR loss (%)	—	49	—	45	48
	coeff. of variation in global CSR	—	1.23	—	1.08	1.15
HadCM3	global mean CSR change (%)	−20.0	−16.6	−2.9	−6.9	−11.6
	regional mean CSR change (%)	42.0	36.8	19.2	24.3	30.3
	global area with CSR loss (%)	51	49	44	46	49
	coeff. of variation in global CSR	1.40	1.28	1.04	1.10	1.18
PCM	global mean CSR change (%)	−0.7	0.6	3.0	2.5	1.4
	regional mean CSR change (%)	20.9	18.0	9.9	12.5	15.1
	global area with CSR loss (%)	42	40	34	36	38
	coeff. of variation in global CSR	0.97	0.96	0.91	0.93	0.94
mean of GCMs	global mean CSR change (%)	−9.4	−8.5	0.3	−2.0	−5.2
	regional mean CSR change (%)	30.9	29.8	15.3	20.0	23.9
	global area with CSR loss (%)	49	49	41	44	47
	coeff. of variation in global CSR	1.04	1.06	0.95	0.99	1.01

Unlike the rather moderate changes in the global average future CSR, the projected changes in regional CSR at an individual grid cell basis are much more pronounced. Absolute changes in regional CSR considered independently from the direction of change are higher, the larger the expected temperature rise. For the B1 scenario, the average CSR change per cell reaches 15.3 per cent, and for the A1FI scenario, there is an average change of 30.9 per cent per grid cell, reaching 42 per cent in the most extreme HadCM3 circulation model (table 2).

The uneven distribution of species richness around the globe is one of the most striking patterns in ecology and biogeography (Hawkins *et al*. 2003; Ricklefs 2004). According to our analysis, the global distribution of CSR will become profoundly more uneven than at present, as evidenced by an increase in the coefficient of variation in the year 2100 compared with today, calculated as the ratio of the standard deviation of all regional CSR values to the global mean CSR (table 2).

We calculated the CSR for the year 2100 based on all 18 available combinations of IPCC scenarios and GCMs. Global CSR declined significantly in 13 of the 18 different models by 2100, on average by 4.9 per cent. To indicate the sensitivity of our results towards differences emerging from different GCMs, we calculated the direction of change and summed up the number of models indicating either increasing or decreasing CSR (figure 2*d*). Among all 18 models, 74 per cent of the land surface showed 100 per cent congruence in the direction of change. Inconsistent results were found in the transition zone between increasing and decreasing CSR, in particular in parts of the Amazon basin and central to southern Africa. The results indicate that independent from the magnitude of the expected future climate change, the direction of the calculated response in terms of CSR changes is similar in most parts of the world. The absolute changes in CSR, however, largely depend on the magnitude of climate change.

When averaged across the 40 industrialized countries listed in the Kyoto Protocol Annex B that are responsible for the highest *per capita* CO_2_ emissions worldwide, the mean CSR for the year 2100 significantly increased by an average of 52 (B1) and 77 (A1FI) species per grid cell (mean CSR today: 594 species; two-sample paired Wilcoxon signed-rank test, *p* < 2.2 × 10^−16^; figure 3*b*). By contrast, the mean CSR decreased significantly by 64 (B1) to 186 (A1FI) species per grid cell (two-sample paired Wilcoxon signed-rank test, *p* < 2.2 × 10^−16^) in countries not listed as industrialized (mean CSR today: 1099 species). This apparent difference is mostly owing to the projected increase in CSR, owing to warming at higher latitudes, whereas CSR in most non-industrialized developing countries is projected to decrease owing to declining water availability. This discrepancy is alarming as the countries richest in plant biodiversity also are projected to experience the largest net loss in CSR. Moreover, it is inequitable that the countries being least responsible for the carbon dioxide concentration in the atmosphere are likely to be confronted with highest biodiversity threat owing to greenhouse gas-induced climate change. This is particularly worrying as the potential to develop climate change mitigation and adaptation strategies is much lower in these countries when compared with industrialized ones (IPCC 2007).

For both the A1FI and the B1 scenario, a pronounced global discrepancy surfaced between regions exposed to either increasing or decreasing CSR (figure 2*b*,*c*). Calculated across 13 major biomes in their current-day spatial location (excluding mangroves as an azonal system; Olson *et al*. 2001), we found that by 2100 CSR shows the highest increase in tundras, followed by boreal forests, temperate coniferous forests, montane grasslands and shrublands, broadleaf and mixed forests and temperate grasslands (figure 3*c*). In these systems, CSR might increase as a result of a relaxation from harsh thermal constraints, such as the occurrence or severity of frost or the duration of the thermal vegetation period, which all strongly limit plant distributions and richness (Sakai & Weiser 1973; Woodward 1987). On the other hand, a decrease in CSR is observed in biomes such as deserts and xeric shrublands, tropical and subtropical dry broadleaf forests, flooded grasslands and savannahs, tropical and subtropical grasslands, tropical and subtropical moist broadleaf forests, as well as in tropical and subtropical coniferous forests. The decrease in CSR in these areas can be explained by a shift of water balance to more negative values and resulting in an excess of drought tolerance levels for many species (compare Baltzer *et al*. 2008; Engelbrecht *et al*. 2007). If the Amazon rainforest is considered independently from African and Asian rainforests, it shows the most severe decrease in CSR compared with all other regions, with losses of approximately 30 (B1) to 50 per cent (A1FI). This corresponds to a potential dieback of Amazon forests by 2100 suggested by some GCMs (Cox *et al*. 2004). Minor CSR changes are projected for temperate grasslands, savannahs and shrublands and in Mediterranean forests, woodlands and scrub. The low effect of climate change on CSR in Mediterranean regions may be explained by not resolving the seasonal distribution of precipitation in the GLM. The ranking of biomes differs slightly when absolute and relative changes in CSR are compared.

Modelled CSR values provide insights into the potential of an area to host a certain number of species. Thus, future CSR projections represent a first baseline risk assessment of the global distribution of plant diversity in the face of climate change. Similar to environmental niche modelling, we employ the covariation of environmental variables and species richness in space to derive temporal predictions (i.e. ‘space-for-time’ substitution; La Sorte *et al*. 2009). An obvious limitation of this approach is that it does not provide direct information about possible range expansions, contractions or extinctions. While the modelled projections account for particular aspects of future climate change, they do not address the complexity of species interactions, potential additional environmental constraints and changes in the non-climatic environmental variables that were not included in the model. Moreover, it is yet unclear how climate-richness relationships vary over time, and whether the same relationships will hold under future climate conditions. Another uncertainty of our approach comes from novel future climate conditions and climatic extremes (Williams *et al*. 2007).

The considered timespan of roughly one century appears too short to trigger substantial speciation events for vascular plants. Short-term changes in local species composition and richness should therefore mostly come about owing to species colonizing from other areas and arise from local extinctions. There is evidence that most species tend to keep their ecological preferences when colonizing new habitats (Crisp *et al*. 2009). For this reason, some regions may lack the appropriate number of suitable species to fill the provided habitat space. Future climate conditions equivalent to current conditions will in many cases be beyond reach owing to geographical distance or may be even non-existent (Williams *et al*. 2007). The risks of climate change-induced range shifts are multiplied in transformed and fragmented landscapes that provide little accessible space, reduced migration routes and little flexibility for the persistence of disadvantaged native species (Walther *et al*. 2002; Svenning & Skov 2004). On the other hand, the spatial patterning of landscape features and environmental variables at different spatial scales can also have a stabilizing effect on species distributions. Many species may be able to persist in small pockets of suitable conditions, e.g. in valleys or gallery forests with still suitable meso- and microscale conditions, even when the overall broad-scale climate conditions are getting harsh and unsuitable.

The rate at which climate is projected to change and at which species displacement is induced in many cases may exceed the velocity at which new arriving species and functional communities are able to establish (Hector *et al*. 1999). Changes in species composition require time for dispersal and recruitment success of invasive species as well as displacement of formerly native species confronted with unfavourable conditions. Disturbances and catastrophic events (Pounds *et al*. 2005) as well as complex biotic interactions (Pearson & Dawson 2003) can further influence the velocity of this process. Ecosystem changes are not likely to appear gradually but are connected to thresholds and tipping points (Scholze *et al*. 2006). In terms of species richness, this can result in timespans with relative stability followed by a cascade of local extinction events. However, as the current occurrence of species represents their realized niches that can be considerably smaller than their fundamental ones (Araújo & Pearson 2005), some species ranges could be considerably more resilient to changing climate conditions than expected. Hence, the eventual achievement of equilibrium between local CSR and realized species richness is subject to interacting factors related to the resilience capacity of individual species and communities (Leemans & Eickhout 2004).

While the negative impacts of a climate-change-induced reduction in regional CSR on global biodiversity and ecosystem functions are apparent, perils of increasing CSR are less obvious at first glance. From a human perspective, an increase in CSR may even be associated with some positive effects such as higher agricultural productivity, higher carbon storage and a wider range of options to manage ecosystem services in some parts of the world (Leemans & Eickhout 2004). On the other hand, a fast increase in CSR beyond the potential for adaptation by established ecosystems may signal high prevalence of species invasions and an extensive replacement of native floras by widespread and competitive species immigrating from elsewhere (Scholze *et al*. 2006; Woodward & Kelly 2008). Paradoxically, an increase in CSR can thereby even cause an intermediate decrease in the absolute species numbers within many regions. Especially, species adapted to harsh environmental conditions may be particularly vulnerable if the climate becomes more favourable for generalists. In this respect, endemic species may get more threatened, as many of them evolved under long-term stable climatic conditions (Jansson 2003; Linder 2008). As a consequence, future climate change may trigger the reallocation of the global pool of existing species. Competitive generalist species will get more abundant and widespread at the expense of specialists that will get more rare and range-restricted or even go extinct, resulting in biotic homogenization (White & Kerr 2007; La Sorte *et al*. 2009). Altogether, this may alter ecological interactions. Although newly arriving species may fill in some of the ecological functions of disappearing species, there is a high risk that ecosystem functions and services may be impaired (Schröter *et al*. 2005).

Our results indicate that the consequences of climate change for plant distributions differ dramatically between the two examined IPCC scenarios, B1 and A1FI. Hence, a precautionary principle dictates that an immediate implementation and continuous further improvement of mitigation strategies are necessary to minimize negative impacts on biodiversity, environmental functionality and sustainable human development. In addition, our results reinforce the necessity of regionalized adaptation strategies in regions with either expected increase or decrease in CSR to minimize the negative impacts of climate change.
